# Working and Learning

**Published:** 1920-03-06

**Authors:** 


THE PUBLIC WELL-BEING—(*continued*)

### Working and Learning.

### Two Aspects of the Education Act.

Some of the firstfruits of Mr. H. A. L. Fisher's great Education Act are near the process of being gathered. One of them is the starting of compulsory day continuation schools for young people of fourteen years of age. The conditions provide that time must be given by employers so that the schools may be attended in the day time, and this prevents the long day of work followed, when the young people are tired, by evening studies. That old method gave with one hand and took away with the other. But now provision is made not only for reasonable conditions of longer education, but for better chances of longer education on systematic lines. For one boy or girl who leaves school at fourteen and continues self-education a hundred bother no more about it, or have too much to do to undertake it, and many will live to be grateful for the compulsory continuation of learning in decent conditions, just as many now live to regret the absence of such opportunities in the past.

The London County Council and the local education authorities have decided to try to open the London day continuation schools on January 1, 1921. The "appointed day" under the Education Act may be fixed some time in October next, and all who have not then reached the age of fourteen will be required to attend the classes from the age of fourteen to the age of sixteen. The attendance required is a minimum period of 320 hours a year, which may be distributed as eight hours a week for forty weeks, but the authorities consider that the most convenient arrangement would be for the young persons to attend the schools for two half-days of four hours each week.

The Chairman of the London County Council Education Committee, in outlining the main points of the measure to a meeting of Press representatives, emphasised the fact that all employers of labour were affected by the Act. Small shopkeepers and mistresses employing maids would have to release their young employees for the required numbers of hours each week. The Council's first group of compulsory day continuation schools will open with about 15,000, and a further 15,000 will be added to the total enrolment every three months. In the London area twenty-two schools will be opened in January 1921, each school providing 360 school places, and the ultimate annual expenditure is estimated at £317,000.

In a report submitted to the London County Council Education Committee, the Higher Education Sub-Committee No. 2 note that in its demands on the working hours of juvenile labour this great new experiment in national education will have far-reaching effects on the industrial and commercial world. It is suggested that the curriculum should be of a general character, and that in the case of young people of from sixteen to eighteen, who will not come within the scope of the obligation until seven years after the "appointed day," the training should have a vocational bias and include the underlying principles of various technical subjects. With shorter hours of labour, one of the great problems will be to train young people to utilise their leisure hours satisfactorily, and for this the schools will endeavour to provide by means, amongst others, of sports and social organisations. Large business houses have already instituted voluntary day continuation schools for their own employees, and the Sub-Committee consider that this phase of the development should be approved during the present year, the firms undertaking to provide suitable accommodation for the purpose free of expense.

**THE PUBLIC WELL-BEING**—(*continued*).

One of the most valuable fruits of the Education Act which remains ungathered is the abolition of the half-time system. No day has been appointed as yet on which Clause 8, which abolishes the half-time system, shall be put in force throughout the country, and in consequence the evil of attempting to work children hard for part of the day and school them for the rest is still rampant. The *Manchester Guardian* says it is true many enlightened authorities have chosen to forestall coercion in this matter, for figures now to hand show that Leeds, Nottingham, Sheffield, and Wigan, among other cities, have now no half-time scholars on their books; while Bradford, Hindley, and Lancaster have creditable decreases to boast of. The recalcitrants, as might be expected, are the rival cotton towns, Blackburn, Burnley, Preston, Oldham, Rochdale, and the rest. It is not failure to appreciate the ultimate value of the reform that holds them back, but a canny determination not to be first in a field that is expected immediately to handicap any that enters it alone. Thus the Rochdale authority—and Rochdale is the greatest sinner of the lot, with an increase in half-timers from 871 in 1915 to 1,219 in 1920—asserts with confidence that "the parents in Rochdale will acquiesce without a murmur in a *national* enforcement; but to abolish half-time in one town and not in another is the way to cause irritation." Many other authorities make the same plea for speedy general action. What (asks the *Guardian*) deters the Government from taking it?

